# Association of American Medical Editors

**Published:** 1879-05-20

**Authors:** R. C. Word

**Affiliations:** Secretary


### *ASSOCIATION OF AMERICAN MEDICAL EDITORS.*

The above Association convened at 8 o'clock p.m., May 5th, in the Senate Chamber, Atlanta, Ga., and was called to order by president W. Brodie, of Detroit, who requested Dr. R. C. Word, of SOUTHERN MEDICAL RECORD, to act as Secretary.

The following gentlemen appeared and registered as members, to wit:

Dr. L. Connor—Detroit Lancet.
Dr. Duncan Eve, Dr. D. J. Roberts—Southern Practitioner.
Dr. Frank Woodbury—Boston Medical and Surgical Journal.
W. P. Jones, M. B.—Southern Journal of Medical and Physical Science, Nashville.
Dr. E. S. Dunster—Michigan Medical News.
Dr. T. S. Powell—Southern Medical Record.
Dr. Theophilus Parvin—American Practitioner.
Dr. W. T. Goldsmith—Southern Medical Record.
Dr. A. N. Bell—The Sanitarian.
Dr. J. G. Westmoreland—Atlanta Medical and Surgical Journal.
Dr. Wm. Brodie—New preparations.
Dr. Robert C. Word—Southern Medical Record.
Dr. Dudley S. Reynolds—Medical Herald, Louisville.
Dr. J. V. Shoemaker—Medical Bulletin.
Dr. Stephen Smith

Dr. Brodie made an able and interesting address on the *History, Scope, Duty and Destiny of American Medical Journals.* Our space will not allow its publication, but we hope that some of the larger journals will publish it.

A resolution suggested by the Speaker, condemnatory of advertising patented and copyrighted nostrums was moved by Prof. Dunster, and elicited remarks from Drs. Davis, Connor, Parvin and others.

The resolution was adopted and referred to American Medical Association.

Dr. Brodie alluded to the illiberality of a certain journal in refusing to exchange with cheaper and smaller journals; whereupon the following resolution, by Dr. Jones, of Nashville, was adopted.

Resolved, That the members of this Association report at the next annual meeting such journals as may decline to exchange with them.

Dr. Parvin made touching allusion to the death of Dr. Hays and Dr. Waddell since last meeting, and moved the appointment of a committee to prepare a paper on the same; adopted :

Committee : Drs. Parvin, Davis and Bell.

Dr. W. T. Goldsmith introduced the following resolution:

Resolved, That the articles of this Association shall be amended to read as follows :

"The Association of American Medical Authors and Editors."

To be acted on at next meeting.

On motion, Dr. Davis, Parvin and Reynolds, were appointed a Committee to Consider Ways and Means for Improving the Interests of the Meetings, and to report at next meeting one or more themes for discussion.

Remarks were made by Drs. Davis, Powell, Woodbury, Goldsmith, Connor, Reynolds and others.

The officers elected for the ensuing year are Dr. S. T. Powell, of Southern Medical Record, Atlanta, Ga., President; and Dr. Frank Woodbury, of Boston Medical Journal, Vice President.

Dr. Powell returned his thanks for the honor conferred, in eloquent and appropriate remarks, and the Association adjourned until day before the meeting of the American Medical Association, in New York, 1880.

R. C. WORD, M.D., Secretary.

