# Correction

**Published:** 1879-06-20

**Authors:** 


### *CORRECTION.*

Our attention has been called to the fact that the Resolution, in the proceedings of the Association of Medical Editors, requiring members to report to the next meeting the names of such Journals as refuse to exchange with them, was introduced by Dr. D. J. Roberts, of Nashville, and not by Dr. W. P. Jones, as published.

